# Mycotoxins: An ongoing challenge to food safety and security

**DOI:** 10.1371/journal.ppat.1013672

**Published:** 2025-11-10

**Authors:** Neriman Yilmaz, Carol Verheecke-Vaessen, Chibundu N. Ezekiel

**Affiliations:** 1 Department of Biochemistry, Genetics and Microbiology, Forestry and Agricultural Biotechnology Institute (FABI), University of Pretoria, Pretoria, South Africa; 2 Magan Centre of Applied Mycology, Cranfield University, Cranfield, United Kingdom; 3 Department of Agricultural Sciences, BOKU University, Institute of Bioanalytics and Agro-Metabolomics, Tulln, Austria; University of Maryland, Baltimore, UNITED STATES OF AMERICA

## Introduction

Mycotoxins have influenced human and animal health for centuries, often with serious and sometimes deadly consequences. The earliest known cases are outbreaks of ergotism in medieval Europe, caused by alkaloids from *Claviceps purpurea* growing on rye. These epidemics, called “St Anthony’s fire”, produced convulsions, gangrene and death. Ergot-infected grain has also been suggested as a possible factor behind the symptoms recorded during the Salem witch trials in 1692 [1]. During the Second World War, people in Russia consumed overwintered grain infected by trichothecene-producing *Fusarium* species. This led to the alimentary toxic aleukia epidemic, one of the best-documented examples of human mycotoxicosis [1]. Such outbreaks demonstrate the longstanding impact of mycotoxins on societies.

The modern era of mycotoxin research began with the “Turkey X disease” outbreak in the United Kingdom in 1960, when contaminated peanut meal caused the deaths of more than 100,000 turkeys. The toxic agents were identified as aflatoxins produced by *Aspergillus flavus* and *Aspergillus parasiticus*, leading to the introduction of the term “mycotoxin” in 1962 [1,2]. Around the same period, sporidesmin A, produced by *Pseudopithomyces toxicarius* (then *Sporidesmium bakeri*), was identified as the causal agent of facial eczema in livestock in New Zealand [3,4]. The first human fatality from the consumption of aflatoxin-contaminated food was documented in Uganda in 1967 as fatal hepatitis [5]. These events initiated systematic research on fungal toxins in agriculture, food safety, and animal health.

Mycotoxin contamination remains a global concern in food and feed systems. Recent outbreaks of aflatoxicosis in dairy herds in Pakistan and fatal maize-related poisoning in Tanzania show that exposure persists in both animals and humans [6,7]. To date, more than 400 mycotoxins have been identified; however, only a few are regulated globally. The major agriculturally significant mycotoxin groups include aflatoxins, trichothecenes, zearalenones, ochratoxins, ergot alkaloids, fumonisins and patulin, produced mainly by *Aspergillus*, *Claviceps, Fusarium* and *Penicillium* species [2]. It is estimated that 60–80% of consumed food contains detectable mycotoxins, and about half of these samples include multiple toxins, forming the so-called “mycotoxin cocktail” [8].

Despite regulatory efforts, mycotoxins remain a major cause of food and feed recalls. Between 2020 and 2024, mycotoxins made up 2,407 notifications (11.6%) in the European Rapid Alert System for Food and Feed, mostly associated with cereals, nuts, and dried fruits imported from tropical regions [9]. Such recalls and rejections have major economic implications for high-, low- and middle-income countries. In Africa, the combined impact of mycotoxin contamination on crop rejection, livestock productivity, and public health has been estimated to cost hundreds of millions of US dollars annually [10]. In the United States, aflatoxin contamination alone has been projected to cause economic losses of up to USD 1 billion per year to the corn industry [11]. Sampling and routine testing also contribute substantially to the overall economic burden, costing the North American agri-food sector more than USD 200 million each year [2]. These studies illustrate how difficult full control remains, even under strict monitoring systems.

Mycotoxin production is controlled by biosynthetic gene clusters that respond to environmental stress factors such as temperature, humidity, water activity and nutrient limitation [2]. Contamination persists because of fungal diversity, environmental stress and agricultural practices. Climate change (CC) is further shifting the range and ecology of toxigenic species [12]. Although better crop management and storage have reduced contamination in some areas, control remains uneven, and many emerging metabolites are still poorly characterised, making risk assessment difficult [12].

**Fig 1:**
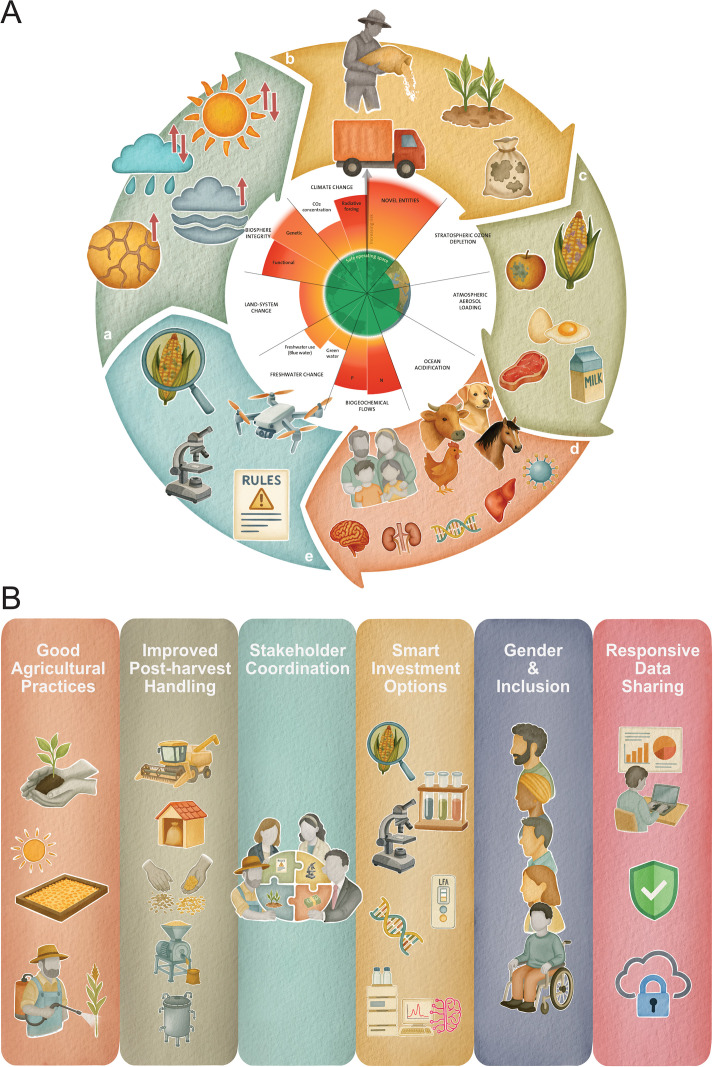
Climate change and planetary boundary interactions driving mycotoxin contamination across the food system (A) and integrated mycotoxin mitigation framework across the crop value chain (B). **(A-top)** This conceptual diagram illustrates the complex, cyclic impact of climate change and planetary boundary stressors on the proliferation of mycotoxigenic fungi and mycotoxin contamination in the crop-food-livestock-human continuum. (a) Climate-related factors such as elevated temperatures, altered rainfall, and drought influence fungal growth and mycotoxin production. (b) Agricultural intensification and fertiliser use affect soil and crop susceptibility to contamination. (c) Contaminated food and feed products enter the food chain. (d) Exposure leads to One Health impacts on humans and animals, including immunosuppression and carcinogenesis. (e) Monitoring and regulatory frameworks using surveillance and risk assessment attempt to mitigate exposure. At the centre, the planetary boundary framework in the centre adapted with credit to Azote for Stockholm Resilience Centre, based on analysis in [44]. It underscores how unsustainable pressures on Earth’s systems amplify risks to food safety. The climate change boundary, already transgressed, measures changes in Earth’s energy balance due to rising greenhouse gas concentrations. Increased atmospheric carbon dioxide traps heat that would otherwise escape to space, driving global temperature rise and altering climate patterns. These shifts create favourable conditions for mycotoxigenic fungi and intensify food safety challenges **(B-bottom):** This framework outlines six coordinated strategies to minimise mycotoxin contamination in food and feed systems. These include: (1) Good agricultural practices (e.g., soil preparation, resistant seeds, safe pest control); (2) Improved post-harvest handling (e.g., drying, sorting, milling, fermentation); (3) Stakeholder coordination to facilitate knowledge sharing and capacity building leading to accurate needs assessment, system sizing and business models; (4) Smart investment options including biotechnology, diagnostics, and AI-assisted tools; (5) Promotion of gender equity and social inclusion; and (6) Responsive data sharing for surveillance and early warning. Together, these interventions aim to sustainably reduce contamination risks, promote food safety, and align with the One Health approach. **Illustration:** Matt Jackson (Vuka Design), created using Procreate and arranged by Neriman Yilmaz using Affinity Publisher 2.

In this review, we address key questions regarding mycotoxins, including identifying the environmental conditions that promote their development, the influence of CC, health impacts from a One Health (OH) perspective, advancements in detection and measurement techniques, and effective strategies to prevent contamination in crops while promoting sustainable solutions that fit within planetary boundaries (Fig 1).

## What are mycotoxins, and why do fungi produce them?

Mycotoxins are secondary metabolites produced by fungi in response to environmental or biological stress, where they can function in defense, competition, and communication. The commonly known mycotoxins include aflatoxins (AFB_1_, AFB_2_, AFG_1_, AFG_2_ and AFM_1_), fumonisins (FB_1_, FB_2_, FB_3_ and FB_4_), trichothecenes (deoxynivalenol (DON), diacetoxyscirpenol, T-2 toxin, HT-2 toxin and nivalenol), citrinin, cyclopiazonic acid, ochratoxins (OTA and OTB), ergot alkaloids, patulin, zearalenone (ZEN), and a group of recently classified emerging mycotoxins such as 3-nitropropionic acid, alternariol, beauvericin, enniatins, moniliformin, sterigmatocystin and tenuazonic acid [13].

Although mycotoxin contamination is often associated with poor storage, many fungi infect crops much earlier, and toxin accumulation can begin in the field. Mycotoxin-producing fungi are broadly classified as field or storage fungi. Field fungi, such as *Fusarium* and *Claviceps*, infect developing grains and floral tissues, producing trichothecenes, fumonisins, ZEN, and ergot alkaloids under humid or temperature-stressed conditions [2]. Their invasion is often associated with plant stress, insect injury, or excessive moisture. In contrast, storage fungi like *Aspergillus* and *Penicillium* grow after harvest on moist or poorly dried and stored grain [2]. However, both genera can also occur in the field, showing that the line between field and storage fungi is not absolute. Contamination can therefore occur at any stage of the supply chain and remains a complex issue to manage.

The question of why mycotoxins are produced has been the subject of extensive research. The natural environment of food crops is characterised by complex ecological interactions between bacteria, fungi, plants and small invertebrates. Mycotoxins are often produced when fungi sense biological competition for resources or abiotic stress, such as changes in water activity, temperature, or aeration. Under such stress conditions, phyto-signals are triggered, to which the fungi respond, causing the release of secondary metabolites as a stress response.

Recent studies have provided evidence that mycotoxins also play ecological roles in defensive or signalling interactions with other microbes or small insects [14]. One of the strongest examples is patulin, and to some degree penicillic acid, which can act as quorum-sensing inhibitors in Gram-negative bacteria by disrupting acyl-homoserine lactone signalling and reducing bacterial coordination and virulence [15,16]. Mycotoxins such as fumonisins and fusarin C have been proposed to function in quorum-like signalling in *F. verticillioides*, influencing interactions with bacterial endophytes and reducing competition [17].

Other mycotoxins have been shown to protect fungi against fungivory or microbial competition. Aflatoxin and its precursor sterigmatocystin give *A. flavus* a selective advantage by deterring insects and suppressing microbial competitors [18]. In feeding trials, the fungivorous springtail *Folsomia candida* preferred *Aspergillus* mutants with reduced mycotoxin production, supporting a defensive role for these metabolites [19]. Similarly, ergot alkaloids produced by *Claviceps* and related endophytic fungi are thought to deter grazing and herbivory [18].

While these examples suggest specific roles for mycotoxins such as defense against competitors, virulence enhancement, and anti-herbivory, the full extent and consistency of their roles in the broader ecological interactions amongst microbes and between fungi and crops remain complex, and it is likely that the reasons for production vary depending on the specific mycotoxin and fungal species.

## Which environmental conditions favour mycotoxin production in fungi, and what role does climate change (CC) play in this process?

Fungi can grow in xerophilic environments as their unique cellular structures and metabolic adaptations allow them to grow on low water activity food (*a*_w_; measure defining the level of water available) food such as 0.647 *a*_w_ [20]. Such food and feed include most of the grains, nuts, spices and dried fruits and vegetables. This ability is subject to multiple influences, including key complementary environmental conditions such as temperature and gas composition, as well as the local microflora and nutrient availability.

Among the genera producing mycotoxins, examples of growth conditions of *Aspergillus* spp. are warmer (6–55 °C) and potentially drier (*a*_w_ ≥ 0.77) [21] compared to *Fusarium* spp. (5–37 °C, *a*_w _≥ 0.88) [22] and *Penicillium* spp. (2–34 °C, *a*_w_ > 0.80) [23]. Permissive growth conditions are broader than the specific conditions required for mycotoxin production, which are often mycotoxin-specific. An example is *A. flavus* production of aflatoxin which only occurred at temperatures of 15–35 °C and *a*_w_ ≥ 0.83 [24]. In case of multiple mycotoxins produced by the same fungi, the production pattern may differ. A recent example is *F. asiaticum* with predicted DON production under a broader range of temperature and a_w_ (5–40 °C; 0.89–0.99 *a*_w_) compared to ZEN production within more restrictive temperature and *a*_w_ ranges (17–37 °C; 0.91–0.99 *a*_w_) [25]

The dynamic environmental conditions within the food supply chain are significantly impacted by CC, which is predicted to increase extreme weather events such as extreme temperatures and associated droughts and floods, with global warming expected to range from 1 to 8.5 °C by the 21st century [26]. These changes are predicted to favour certain mycotoxigenic fungi and increase the risk of specific mycotoxins in different regions. Another crucial aspect of CC is the rising concentration of atmospheric carbon dioxide (CO_2_). A first element of response highlights a potential adaptation of the *Fusarium* spp. to increased CO_2_ levels [27]. CC-related stresses such as drought or heat can weaken plant defence mechanisms, making them more susceptible to fungal colonisation and subsequent mycotoxin production. It is also likely that the weakening of crop resistance due to CC will promote colonisation by opportunistic fungi such as *A. flavus*, which has shown increased aflatoxin production at 37 °C versus 30 °C when exposed to higher CO_2_ levels [28]. CC, including elevated CO_2_, is therefore driving a significant shift in mycotoxigenic fungi and their associated mycotoxins, raising new risks for global food security.

CC impacts mycotoxigenic fungi and mycotoxins globally. In northern and central Europe, heavier rainfall has increased *Fusarium graminearum* contamination, leading to higher DON levels in wheat [29]. This pattern extends to lower-latitude countries like France and Romania, where DON outbreaks have been reported since 2010 [29]. Co-contamination with mycotoxins such as ZEN and T-2 is also on the rise, pointing to shifts in *Fusarium* populations [12,30]. Meanwhile, warming temperatures in southern Europe favour *A. flavus*, increasing aflatoxin risks in maize [12,30]. In South Africa, recent studies report frequent co-occurrence of multiple mycotoxins in diseased maize, along with changes in fungal communities compared to earlier findings [31,32]. Despite these findings, large parts of the world remain under-researched, highlighting the urgent need for global monitoring and proactive food safety strategies.

## How do mycotoxins threaten human and animal health from a One-Health (OH) perspective?

The OH concept, introduced by the World Health Organization (WHO), is defined as “an integrated, unifying approach that aims to sustainably balance and optimise the health of people, animals, and ecosystems”. This framework is particularly relevant in addressing mycotoxin contamination, which poses risks to human and animal health as well as the environment. The WHO has estimated that more than half a billion people, consisting mostly of those living in economically developing regions such as sub-Saharan Africa, are chronically exposed to hazardous mycotoxin levels [10].

Mycotoxicosis, diseases caused by mycotoxins, were mostly overlooked until the 1960s. This period marked a turning point when aflatoxins were identified as potent liver toxins, bringing these diseases into focus as major health concerns [2]. Mycotoxin exposure can be acute, causing nausea and immune suppression, or chronic, leading to organ damage, immune dysfunction and several cancers. Aflatoxins, classified as Group 1 carcinogens by the International Agency for Research on Cancer (IARC), play a significant role in hepatocellular carcinoma cases worldwide. They may be responsible for 4.6% to 28.2% of cases, contributing up to 155,000 cases annually, particularly in regions with high hepatitis B prevalence and concomitant food contamination, such as sub-Saharan Africa, Southeast Asia, and China [33]. Fumonisins, classified as Group 2B carcinogens by the IARC, have been linked to liver cancer, with possible associations with oesophageal cancer [34], while trichothecenes suppress the immune system [35].

In animals, mycotoxin-contaminated feed impairs growth, fertility, and immunity, leading to economic losses and potential toxin transfer into animal products. Aflatoxins cause liver damage in livestock and poultry, while OTA harms kidney function in pigs and poultry [2]. Fumonisins trigger neurological and respiratory disorders, while ZEN disrupts reproduction in pigs [2]. Beyond the regulated mycotoxins, others such as sporidesmin A, patulin, moniliformin, sterigmatocystin, and citrinin cause severe health issues ranging from gangrene and reproductive problems to kidney and liver toxicity and gastrointestinal disturbances [2,36].

From an OH perspective, mycotoxin contamination not only threatens human and animal health but also impacts food security and the environment. Contaminated crops lead to economic losses, disproportionately affecting low-income regions. For example, large quantities of contaminated feed may need to be disposed of, creating environmental waste management challenges. Furthermore, the impact of fungal growth on crop yields can lead to increased land use pressures as farmers seek to compensate for losses. CC further exacerbates the mycotoxin menace, thereby increasing mycotoxin risks in food and feed.

## What are the most effective methods for detecting and accurately measuring mycotoxin contamination in food?

Given the significant health risks posed by mycotoxins, several global or regional organisations, including the European Food Safety Authority (EFSA), the US Food and Drug Administration (FDA), Codex Alimentarius, the Joint Food and Agriculture Organization (FAO)/WHO Expert Committee on Food Additives (JECFA), have established maximum permissible limits in food and feed, reinforcing the need for reliable detection methods [37,38]. Analytical techniques including liquid chromatography tandem–mass spectrometry (LC–MS/MS), high-performance liquid chromatography, and gas chromatography–mass spectrometry provide high specificity and quantification accuracy. Among these, LC–MS/MS has become the gold standard due to its sensitivity and ability to quantify over 700 mycotoxins and secondary metabolites in complex matrices [39].

Recent advances have shifted toward integrated systems that combine analytical precision with portability and automation. Lateral flow immunoassays, similar to pregnancy tests, use specific antibodies for qualitative detection, offering portability and rapid results. Biosensors with electrochemical, optical, or molecular-based detection systems provide real-time monitoring with high sensitivity. Non-invasive optical techniques, such as hyperspectral imaging, fluorescence, and infrared spectroscopy, can detect compositional changes associated with fungal growth but typically identify indirect indicators rather than mycotoxins themselves.

The field of mycotoxin detection is continuously evolving, with a clear trend towards integrating advanced analytical techniques, rapid screening methods, and Artificial Intelligence (AI)-driven tools for more efficient and comprehensive monitoring of mycotoxins. The integration of LC–MS/MS-based screening, biosensors, and AI-assisted workflows promises to transform mycotoxin detection. Specifically, AI-driven hyperspectral imaging and machine learning can enhance automated contamination [38,40]. In addition to improving efficiency, the integrated detection methods will challenge and redirect the status quo of food safety monitoring for regulatory compliance.

## What strategies can be adopted to minimise or prevent mycotoxin contamination in crops?

Over the years, several seemingly fragmented approaches have been proposed and tested towards mycotoxin control in food crops, with some substantial investments disseminated into their implementation. However, the complexity of the global mycotoxin challenge continues to restrict optimum results, posing a wider challenge especially among the economically developing regions where precarious contamination levels occur. An understanding of the intertwined hurdles that span several domains (e.g., biological, agriculture, health, socioeconomics and policy) is required to propose effective control strategies.

Despite the efforts, it suffices to note that there is no silver bullet strategy to control mycotoxins in crops due to the myriad of climatic, biological, social and economic development factors that influence mycotoxin contamination in crops. Accordingly, adopting an integrated and multi-pronged approach to mycotoxin control has been proposed as the most promising strategy to curb this global menace [10]. This includes sustainable scientific and non-scientific options that cover the entire crop value chain and involve multi-stakeholders such as: (1) crop-tailored good agriculture practices (e.g., adequate land preparation at pre-planting, sowing improved seed varieties, timely weeding, application of crop growth enhancers and safe pest control agents, plant health monitoring, timely harvesting of crops); (2) combined simple and improved postharvest crop handling practices (e.g., drying to safe moisture levels in controlled environments, hand- and optical sorting, grain cleaning, dry milling, nixtamalisation, fermentation and cold-plasma); (3) systematic and coordinated activities of crop value chain stakeholders that result in enhanced and targeted sensitisation of relevant actors, skill empowerment for safe food production; (4) accountability-based smart investment options (e.g., government-development agency-private sector triad partnerships, incentivisation of stakeholders at the primary producer levels including the most vulnerable populations, and capacity-building for field testing and monitoring of crop contamination); (5) promotion of gender equality and social inclusion across all levels of stakeholders and (6) responsible data sharing [41–43]. Implementing these preventive strategies not only protects human and animal health by reducing exposure to harmful mycotoxins, but also contributes to food security by minimising crop losses and reducing economic drain-offs for farmers and the agricultural sector, aligning with the principles of OH. The efficacy of this suggested integrated approach, combined into a mycotoxin mitigation tool-kit, should be evaluated in well-designed, multi-location, multi-year research studies.

From an OH perspective, effective mycotoxin risk management requires coordinated efforts between agriculture, veterinary and public health sectors to improve detection, reduce contamination and enhance food safety. Preventive measures, such as better storage, biocontrol strategies and the development of resistant crop varieties, play a critical role in limiting exposure. Strengthening research capacity and improving risk assessment are key to developing sustainable, locally relevant solutions. These efforts will build long-term resilience, protect public health, support ecosystems and secure food systems amid growing environmental and socio-economic challenges while fitting into the planetary boundaries.

## Conclusion remarks

In conclusion, mycotoxins represent an ongoing and complex challenge to food security, public health and the overall economy. Factors such as evolving fungal populations, the impacts of CC and the widespread occurrence of co-contamination underscore the persistent threats posed by these toxins. Addressing this global issue effectively requires an integrated and multi-pronged approach, encompassing good agricultural practices, improved postharvest handling, stakeholder collaboration, smart investments, gender equality and social inclusion, and responsible data sharing across the entire crop value chain. Given the absence of a single solution, such a comprehensive strategy is crucial for minimising mycotoxin contamination, safeguarding human and animal health through an OH perspective, and ensuring global food security.
